# The Protective Effect of Echinochrome A on Extracellular Matrix of Vocal Folds in Ovariectomized Rats

**DOI:** 10.3390/md18020077

**Published:** 2020-01-24

**Authors:** Ji Min Kim, Jeong Hun Kim, Sung-Chan Shin, Gi Cheol Park, Hyung Sik Kim, Keunyoung Kim, Hyoung Kyu Kim, Jin Han, Natalia P. Mishchenko, Elena A. Vasileva, Sergey A. Fedoreyev, Valentin A. Stonik, Byung-Joo Lee

**Affiliations:** 1 Pusan National University School of Medicine and Medical Research Institute, Pusan National University, Yangsan 50612, Korea; ny5thav@hanmail.net; 2 Biomedical Research Institute, Pusan National University Hospital, Busan 49241, Korea; kmky82@naver.com; 3 Department of Otorhinolaryngology-Head and Neck Surgery and Biomedical Research Institute, Pusan National University Hospital, Busan 49241, Korea; cha-nwi@daum.net; 4 Department of Otolaryngology-Head and Neck Surgery, Samsung Changwon Hospital, Sungkyunkwan University School of Medicine, Changwon 51353, Korea; uuhent@gmail.com; 5 Department of Life Science in Dentistry, School of Dentistry, Pusan National University, Yangsan 50612, Korea; hskimcell@pusan.ac.kr; 6 Institute for Translational Dental Science, Pusan National University, Yangsan 50612, Korea; 7 Department of Nuclear Medicine and Biomedical Research Institute, Pusan National University Hospital, Busan 49241, Korea; buisket@naver.com; 8 National Research Laboratory for Mitochondrial Signaling, Department of Physiology, College of Medicine, Cardiovascular and Metabolic Disease Center (CMDC), Inje University, Busan 47391, Korea; estrus74@gmail.com (H.K.K.); phyhanj@inje.ac.kr (J.H.); 9 G.B. Elyakov Pacific Institute of Bioorganic Chemistry, Far-Eastern Branch of the Russian Academy of Science, 690022 Vladivostok, Russia; mischenkonp@mail.ru (N.P.M.); vasilieva_el_an@mail.ru (E.A.V.); fedoreev-s@mail.ru (S.A.F.); stonik@piboc.dvo.ru (V.A.S.)

**Keywords:** echinochrome A, estradiol, extracellular matrix, vocal fold, ovariectomy

## Abstract

Here, we investigated the effects of sex hormones on extracellular matrix (ECM)-related gene expression in the vocal fold lamina propria of ovariectomized (after ovary removal) rats and verified whether echinochrome A (ECH) exerts any therapeutic effects on ECM reconstitution after estrogen deficiency in ovariectomized rats. Sprague–Dawley female rats (9 weeks old) were acclimatized for a week and randomly divided into three groups (*n* = 15 each group) as follows: group I (sham-operated rats, SHAM), group II (ovariectomized rats, OVX), group III (ovariectomized rats treated with ECH, OVX + ECH). Rats from the OVX + ECH group were intraperitoneally injected with ECH at 10 mg/kg thrice a week after surgery for 6 weeks. And rats were sacrificed 6 weeks after ovariectomy. Estradiol levels decreased in OVX group compared with the SHAM group. ECH treatment had no effect on the levels of estradiol and expression of estrogen receptor β (ERβ). The evaluation of ECM components showed no significant changes in elastin and hyaluronic acid levels between the different groups. Collagen I and III levels were lower in OVX group than in SHAM group but increased in OVX + ECH group. The mRNA levels of matrix metalloproteinase (MMP)-1, -2, -8, and -9 were significantly higher in the OVX group than in the SHAM group, but decreased in the OVX + ECH group. Thus, changes were observed in ECM-related genes in the OVX group upon estradiol deficiency that were ameliorated by ECH administration. Thus, the vocal fold is an estradiol-sensitive target organ and ECH may have protective effects on the ECM of vocal folds in ovariectomized rats.

## 1. Introduction

Sex hormones are major factors that influence the activity of the vocal fold and voice production. Voice, a secondary sex characteristic, varies between males and females and changes with sexual maturation. Female voice is closely related to changes in female sex hormones. Laryngeal changes are evident and systematically fluctuate during the reproductive years with the menstrual cycle and affect voice changes throughout life [1,2].

In general, voice production relies on the extracellular matrix (ECM) of the connective tissue in the vocal fold. The ECM of the vocal fold lamina propria comprises interstitial proteins, such as collagen, elastin, and fibronectin, and glycosaminoglycans, such as hyaluronic acid. These ECM components have important biochemical functions and contribute to vocal fold function. These molecules play an important role in determining vocal fold viscoelasticity and affect clinical voice functions, such as phonation threshold pressure and vocal fundamental frequency [3,4,5].

During menopause, the decrease in the amount of estrogen causes dysfunctions of the connective tissues via ECM degradation. Previous studies have shown that the vocal fold is an estradiol-sensitive target organ and any decrease in estradiol levels may affect the expression of several ECM-related molecules in the vocal fold [6]. Hormone therapy is commonly used for the treatment of several menopausal symptoms and for the alleviation of menopausal voice complaints, but adverse-effects of hormone therapy have been reported [7,8,9]. Thus, here we investigated the mechanism underlying improvement in voice quality after menopause using a natural component of marine products.

**Figure 1:**
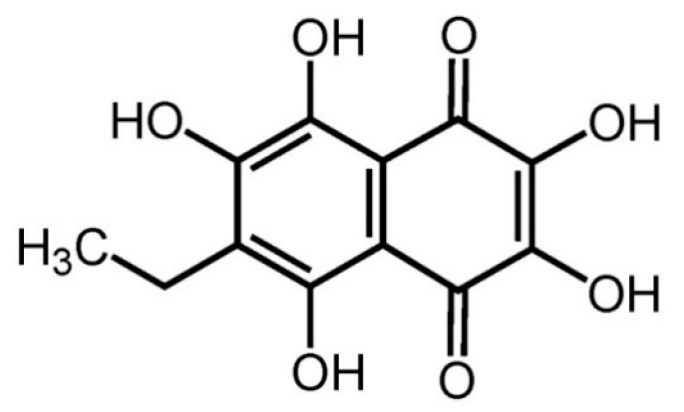
Chemical structure of echinochrome A.

Echinochrome A (ECH, 6-ethyl-2,3,5,7,8-pentahydroxy-1,4-naphthoquinone) is extracted from sea urchins (*Saphechinus mirabilis*) (Figure 1). Its chemical structure is appropriate for free-radical scavenging and includes 2,3,7-hydroxl groups, which are known for their antioxidant properties via regulation of mitochondrial biosynthesis [10]. Studies have shown that antioxidants improve ECM composition, suppress vocal fold wounds, and prevent inflammatory responses [11,12]. Thus, the antioxidant effects of ECH may be expected to play a role in improving the function of vocal fold ECM. Moreover, ECH also contains a 1,4-naphthoquinone ring, which is similar to that observed in vitamin K [13]. In postmenopausal women, vitamin K is reported to reduce menopausal symptoms; however, these studies are limited to bone metabolism or cardiovascular dysfunction [14]. Therefore, ECH may possibly alleviate menopausal symptoms owing to its structural similarity with vitamin K.

To date, studies on vocal folds have focused on vocal fold aging and mechanisms of wound healing. However, reports on sex hormone-specific female vocal aging are scarce. Moreover, the existing reports on female vocal fold aging are mainly morphological studies of the vocal fold of elderly women and the underlying cellular mechanisms of sex hormone-induced vocal fold changes are yet unknown. Thus, the purpose of the present study was to investigate the effects of sex hormones on the alteration of the expression of ECM-related genes in the vocal fold lamina propria of ovariectomized female rats (after surgical ovary removal) and to verify whether ECH exerts any preventive effects on ECM reconstitution following estradiol deficiency in ovariectomized female rats.

## 2. Results

### 2.1. Effect of ECH on Serum Estradiol Level and Expression of Estrogen Receptor β

**Figure 2:**
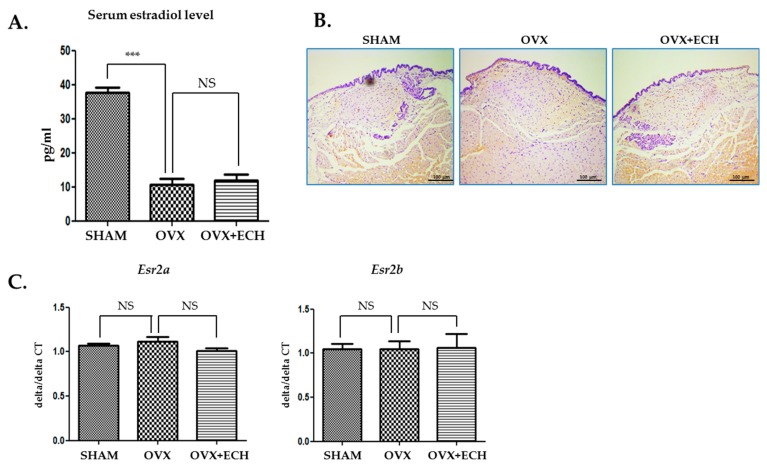
Serum estradiol levels and expression of estrogen receptor β. The level of serum estradiol decreased in the ovariectomized rat, OVX group compared with the SHAM group. ECH treatment did not affect serum estradiol level (**A**). Immunohistochemistry (IHC) staining analyses of representative estrogen receptor β (ERβ) in the lamina propria of vocal folds. The immune-positive area for ERβ was not changed between groups (**B**). Quantitative polymerase chain reaction (qPCR) analyses of genes encoding representative *Esr2a* and *Esr2b* in the lamina propria of vocal folds. The expression of *Esr2a* and *Esr2b* was not changed between groups. The scale bar in each panel is equal to 100 μm (40× magnification). One-way ANOVA test; NS not significant and *** *p* < 0.001 vs. SHAM.

Figure 2A shows the changes in the serum level of estradiol. The level of serum estradiol decreased in the OVX group (10.82 ± 4.35 ng/mL, *p* < 0.001 vs. SHAM) compared with that reported in the SHAM group (10.82 ± 4.25 ng/mL); however, estradiol concentration in the OVX group was similar to that reported in the OVX + ECH group (11.34 ± 5.56 ng/mL). To evaluate the effect of sex hormone on sex hormone receptors, we performed immunohistochemistry and quantitative polymerase chain reaction (qPCR) analyses to determine the expression levels of two isoforms, *Esr2a* and *Esr2b*, in vocal fold lamina propria. ERβ expression was similar between all groups (Figure 2B). According to our previous research, *Esr1* expression was undetected on the lamina propria of female vocal fold with qPCR [6]. Hence, we evaluated the expression levels of *Esr2a* and *Esr2b* using qPCR. The expression level of *Esr2a* and *Esr2b* showed no significant differences between all groups (Figure 2C). Ovariectomy led to a significant decrease in the amount of estradiol and ECH had no effect on both serum estradiol level and ERβ expression.

### 2.2. Effect of ECH on Hyaluronic Acid and Hyaluronic Acid Synthase (Has1, Has2, Has3)

**Figure 3:**
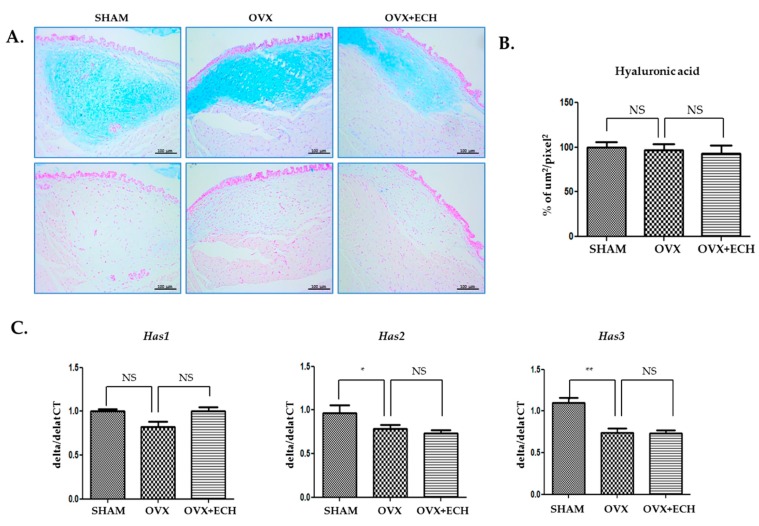
Hyaluronic acid of vocal fold lamina propria. (**A**) Alcian blue stained staining analyses and (**B**) the densities of hyaluronic acid in the lamina propria of vocal folds. The concentration of hyaluronic acid was not changed between groups. Quantitative polymerase chain reaction (qPCR) analyses of genes encoding representative hyaluronic acid synthase (*Has*) 1, 2, and 3 in the lamina propria of vocal folds. The expression of *Has1* was not changed between groups. The expression of *Has2* and *Has3* decreased significantly in the OVX group compare with the SHAM group, but ECH treatment had no effect. The scale bar in each panel is equal to 100 μm (40× magnification). One-way ANOVA test; NS not significant and * *p* < 0.05, ** *p* < 0.01 vs. SHAM.

We determined the concentration of hyaluronic acid with Alcian blue staining. The blue stain was decolorized following hyaluronidase digestion. Hyaluronic acid appeared to be evenly distributed throughout the vocal fold and no significant difference was observed between all groups (Figure 3A,B). The OVX and OVX + ECH groups showed no significant changes in the expression levels of the hyaluronic acid synthase 1 (*Has1*) gene in qPCR. Hyaluronic acid synthase 2 (*Has2*) and 3 (*Has3*) gene levels decreased in the OVX group but failed to change in the OVX + ECH group (Figure 3C). The results confirmed no statistical difference in the total density of hyaluronic acid between different groups. Six weeks after ovariectomy, the expression levels of *Has2* and *Has3* were affected; however, no significant difference was observed in the expression level of *Has1*. ECH treatment had no effect on hyaluronic acid level at 6 weeks after ovariectomy.

### 2.3. Effect of ECH on Collagen and Procollagen (Col1a1, Col1a2, Col3a1)

**Figure 4:**
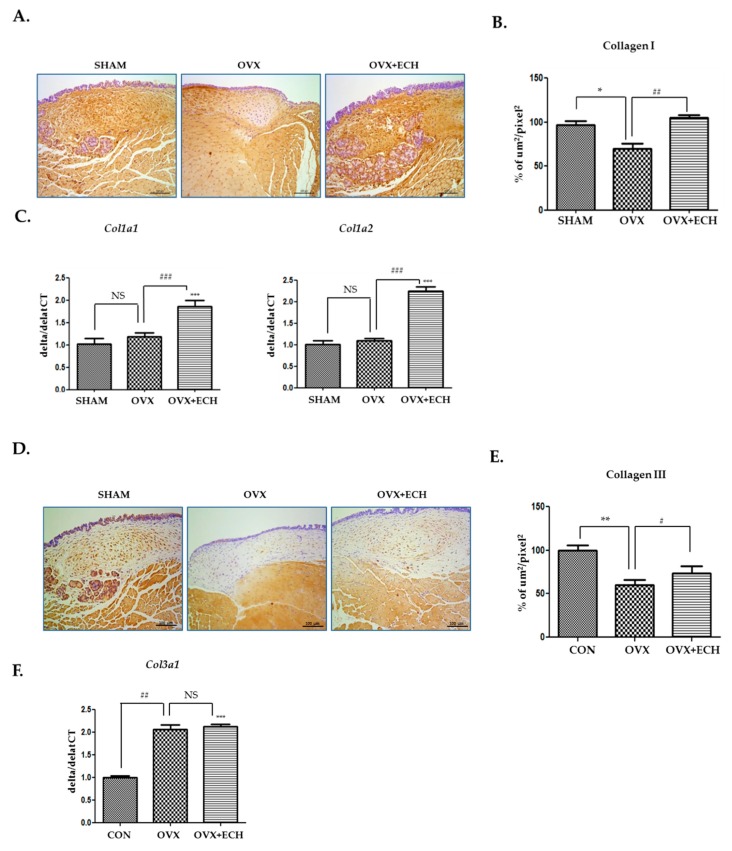
Collagens I and III of the vocal fold lamina propria. Immunohistochemistry (IHC) staining analyses of representative (**A**) collagen I and (**B**) the densities of collagen I in the lamina propria of vocal folds. The immune-positive area for collagen I was decreased in the OVX group and increased in the OVX + ECH group. (**C**) Quantitative polymerase chain reaction (qPCR) analyses of genes encoding representative procollagen *Col1a1* and *Col1a2* in the lamina propria of vocal folds. The expression of *Col1a1* and *Col1a2* did not change in the OVX group compared with the SHAM group, but was significantly elevated in the OVX + ECH group. IHC staining analyses of representative (**D**) collagen III and (**E**) the densities of collagen III in the lamina propria of vocal folds. The immune-positive area for *Col3a1* was decreased in the OVX group and increased in the OVX + ECH group. (**F**) qPCR analyses of genes encoding representative procollagen *Col3a1* in the lamina propria of vocal folds. The expression of *Col3a1* increased in the OVX group compared with the SHAM group, but there was no change in the OVX + ECH group. The scale bar in each panel is equal to 100 μm (40× magnification). One-way ANOVA test; NS not significant, * *p* < 0.05, ** *p* < 0.01 and *** *p* < 0.001 vs. SHAM; # *p* < 0.05, ## *p*< 0.01 and ### *p* < 0.001 vs. OVX.

Collagen I and III are the major types of collagen in the vocal fold lamina propria of rats. The density of collagen I, as evident from immunohistochemical staining, was lower in the OVX group (27.6%, *p* < 0.05 vs. SHAM) than in SHAM group (Figure 4A,B). The decrease in collagen I expression in the OVX group was restored to the level detected in the SHAM group after ECH treatment. The mRNA expression level of procollagen was confirmed with qPCR and no significant difference was observed in the expression levels of *Col1a1* and *Col1a2* between the OVX and SHAM groups. However, the expression levels of *Col1a1* and *Col1a2* increased in the OVX + ECH treatment group compared with those in the OVX group, which were higher than the levels observed in SHAM group (Figure 4C). Collagen III deposition showed a pattern similar to that of collagen I. The density of collagen III in the stained areas from the OVX group was lower than that in the stained areas of the SHAM group. The expression of collagen III in the OVX + ECH group increased compared with that in the OVX group (Figure 4D,E). The expression level of *Col3a1* was confirmed with qPCR. *Col3a1* gene expression was significantly upregulated in the OVX group compared to that in SHAM group, but its expression in the OVX + ECH group was similar to that reported in the OVX group (Figure 4F). These results reveal that ECH treatment increases the protein expression of collagen I via upregulation in the expression levels of *Col1a1* and *Col1a2*. However, mRNA and protein levels of collagen I and III in the OVX group were partially inconsistent. Hence, we confirmed the expression of matrix metalloproteinases (MMPs).

### 2.4. Effect of ECH on Two Elastin Genes (*Eln* and *Cela1*)

**Figure 5:**
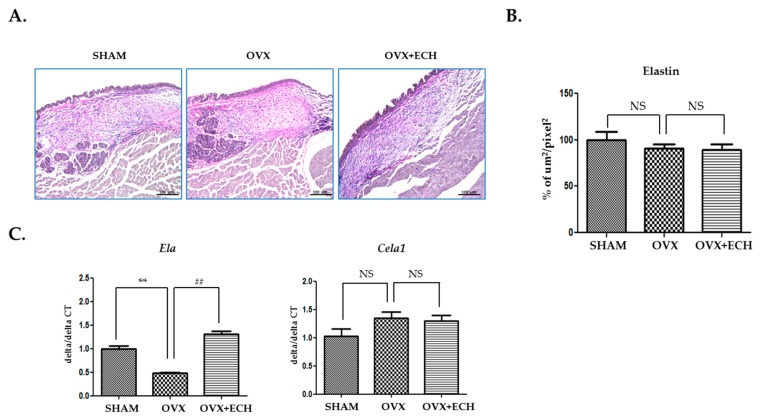
Elastin of vocal fold lamina propria. (**A**) Van Gieson stained staining analyses and (**B**) the densities of elastin fibers in the lamina propria of vocal folds. The expression of elastin was not changed in each group. Quantitative polymerase chain reaction (qPCR) analyses of genes encoding representative *Eln* and *Cela1* in the lamina propria of vocal folds (**C**). The expression of *Eln* decreased significantly in the OVX group compared with the SHAM group, but ECH treatment increased *Eln* level. The expression of *Cela1* was not changed in each group. The scale bar in each panel is equal to 100 μm (40× magnification). One-way ANOVA test; NS not significant; ** *p* < 0.01 vs. SHAM and ## *p* < 0.01 vs. OVX.

To investigate the expression level of elastin, Verhoeff’s elastin staining technique was used. Immunohistochemical staining revealed slightly lower elastin intensity for the OVX group than for the SHAM group, but no statistical significance was reported. The OVX + ECH group showed no change in elastin expression (Figure 5A,B). We examined the expression levels of *Eln* and *Cela1* with qPCR. *Eln* tropoelastin is a precursor of elastin and assembles into elastic fibers. The expression of *Eln* decreased in the OVX group compared with that in the SHAM group, but increased with ECH treatment to the level reported in the SHAM group. The expression level of *Cela1* elastase, an elastin-degrading enzyme, also slightly increased in the OVX group without any statistical significance; ECH treatment had no effect on *Ela* expression level (Figure 5C) but affected the expression level of *Cela1*; however, no significant difference was observed in the expression levels of elastase and immunohistochemical elasatin proteins in elastic fibers.

### 2.5. Effect of ECH on Matrix Metalloproteinases (MMPs)

**Figure 6:**
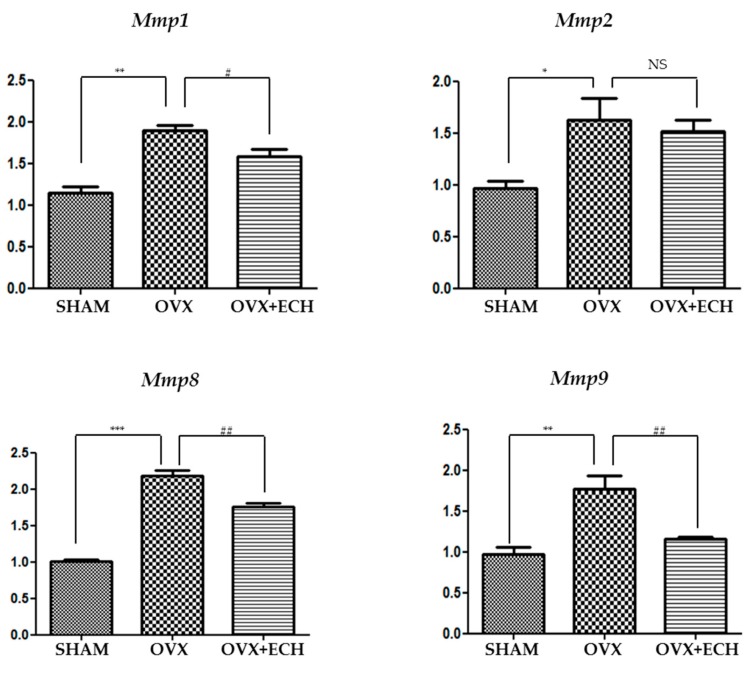
Matrix metalloproteinases in vocal fold lamina propria. Quantitative polymerase chain reaction (qPCR) analyses of genes encoding representative matrix metalloproteinases (*Mmp1*, *Mmp2*, *Mmp8*, *Mmp9*) in the lamina propria of vocal folds. The expression of the matrix metalloproteinases MMPs increased significantly in the OVX group compare with the SHAM group, but ECH treatment decreased MMP levels. One-way ANOVA test; NS not significant; * *p* < 0.05, ** *p* < 0.01 and *** *p* < 0.001 vs. SHAM; # *p* < 0.05 and ## *p* < 0.01 vs. OVX.

We tested the expression levels of *Mmp1*, *Mmp2*, *Mmp8*, and *Mmp9* to determine any inconsistencies between the protein and mRNA levels of collagen (Figure 4). Some MMPs act as collagenase and are involved in the pathological remodeling of tissues. We screened the expression of the genes encoding interstitial MMPs (*Mmp1*, *Mmp8*) and gelatinase MMPs (*Mmp2*, *Mmp9*) in each group with qPCR. The expression level of *Mmp1* and *Mmp8* significantly increased in the OVX group compared with that in the SHAM group, but ECH treatment resulted in a decrease in *Mmp1* and *Mmp8* expression levels (Figure 6). Moreover, the expression level of *Mmp2* and *Mmp9* was significantly higher in the OVX group than in the SHAM group and ECH treatment ameliorated this effect (Figure 6). The expression of MMPs in the OVX group was significantly higher than that in the SHAM group, but ECH treatment reduced these upregulated levels. Thus, *Mmp1*, *Mmp2*, *Mmp8*, and *Mmp9* expression levels increased at 6 weeks after ovariectomy, which resulted in collagen degradation and consequently decreased the expression of collagen, while ECH treatment suppressed collagen degradation by reducing the levels of MMPs.

## 3. Discussion

Changes in sex hormones with menopause affect the female voice. Female sex hormones affect the larynx and vocal folds, thereby influencing voice production. The most drastic change in female voice occurs during menopause. After menopause, women show restricted vocal frequency perturbation range and lower habitual fundamental frequency [15]. These changes may affect social life, especially for professional singers. According to previous reports, the decrease in estradiol levels may cause changes in the connective layers of the vocal fold in postmenopausal women [16]. In addition, estradiol deficiency may cause atrophy of laryngeal muscles and stiffening of laryngeal cartilage. Our previous study evaluated voice changes induced by sex hormones [17]. We have previously reported the effects of ECM production and degradation on vocal fold lamina propria in ovariectomized rats [6]. Population surveys show that many women report voice deepening after menopause, but this observation was not common among men of similar age [18]. This result is also consistent with our previous study. We investigated the changes in the expression of ECM-related genes in orchiectomized (after testis removal) male rats and detected no significant changes. These results suggest that the lamina propria of the vocal fold is an estradiol-sensitive target organ.

Loss of ovarian function after menopause decreases the biological functions of several organs, usually owing to estrogen deficiency. Physical changes caused by menopause include abdominal fat accumulation, osteoporosis, cardiovascular disease, and dry mouth. Thus, estrogen replacement therapy, designed to alleviate menopausal symptoms, may be helpful for menopausal voice therapy [19,20]. Hormone therapy has long been used for the treatment of several menopause-related complaints. Estrogen replacement therapy may alleviate menopause-related voice complaints [21,22]. Vocal quality changes are common in women after estrogen replacement therapy in response to the management of menopause-related conditions. Estrogen replacement therapy has been used to forestall menopause-associated voice changes, especially among professional singers. Cruso et al. showed that the larynx possesses receptors for ovarian sex hormones and that estrogen plays an important role in laryngeal tropism [3]. These previous evidences suggest that estrogen replacement therapy may provide prevention, and possibly treatment, of pathophysiologies, such as vocal cord dystrophy, which are common among postmenopausal women. However, the safety of estrogen replacement therapy is still controversial. Therefore, non-hormonal alternatives, including mineral and vitamin supplements, phytoestrogens, natural hormones, and phytochemicals, are being investigated [23]. In particular, the pharmacological mechanism underlying the effects of isoflavones, the most studied flavonoids, is known. Isoflavones exert antioxidant effects and alleviate menopause-related complications [24,25,26]. Therefore, ECH, a natural marine substance with strong antioxidant properties, may be potentially useful to improve menopausal symptoms. In this direction, we used ECH to verify any changes in vocal cord and voice caused by menopause. 

Oxidative stress is related to voice changes caused by vocal scar and aging [27,28,29]. Antioxidants have been used to prevent vocal fold scars by improving vocal fold wound healing [11,12]. A study was conducted to improve voice after aging [30,31]. However, no studies have been directed to improve voice quality and the cellular mechanisms underlying the effect of these antioxidants on vocal fold lamina propria in menopause are unknown. Therefore, to better understand this menopause-related vocal fold change, we analyzed the preventive effects of ECH on the distribution and production of ECM within the lamina propria of the vocal fold in ovariectomized rats.

ECH is one of the several spinochromes in sea urchins. It has a naphthazarin fragment, which makes it suitable for metal ion chelation. The chemical structure of ECH is appropriate for free-radical scavenging, owing to the presence of 2,3,7-hydroxyl groups. Hence, the antioxidant properties of ECH are related to the regulation of mitochondrial biosynthesis [32,33]. ECH exerts several biological effects. In animal experimental models, ECH reduced glucose concentration, lipid peroxidation, and activities of arginases, such as alanine aminotransferase (ALT), aspartate aminotransferase (AST), alkaline phosphatase (ALP), and gammaglutamyl transpeptidase (γ-GT). Moreover, ECH increased levels of insulin, nitric oxide, and endogenous antioxidant enzymes in the rat liver [34]. In addition, ECH exhibited protective effects and reduced infarct size by up to 45% in a myocardial ischemia/reperfusion injury model by enhancing mitochondrial functions [35]. It is effective against peptic ulcer-induced oxidative stress in rats and this effect is mediated via alleviation of gastric acidity [36]. However, so far, no study has explored the effects of strong antioxidant properties and biological activities of ECH on the vocal fold. Thus, here we investigated the effects of sex hormones on the alteration in the expression of ECM-related genes of vocal fold lamina propria in ovariectomized rats and verified whether ECH exerts any preventive effects on ECM reconstitution following estradiol deficiency in ovariectomized rats. Ovariectomized rats are widely used for the study of prevention and treatment strategies for postmenopausal women because the rat ovariectomized state closely mimics the hormonal conditions of postmenopausal women [37,38].

Analysis of ECM components at 6 weeks after ovariectomy revealed no changes in hyaluronic acid concentration and elastin levels. Hyaluronic acid is one of important components of the ECM and contributes to the viscoelastic properties of the vocal fold lamina propria [39]. Elastic fibers serve as scaffolds for structural maintenance and provide tensile strength and resilience [40]. The expression levels of *Has 2, 3* and *Eln* were slightly different, but the changes in hyaluronic acid and elastin in vocal fold lamina propria were insignificant. These findings differed in part from our previous findings at 12 weeks after ovariectomy [6]. The change in ECM owing to the reduction in estradiol seems to be somewhat different over time.

Collagen level decreased in the OVX group, but the down-regulated collagen level was increased by ECH treatment. Decreased collagen level in the lamina propria correlated with stiffness that disturbed the pliable mucosal wave. Collagen I is ubiquitous and mainly appears as fibrillary bundles. It provides high-tensile strength [39,40]. Collagen III is present in most tissues that require flexibility and elasticity [40,41]. The OVX group showed downregulated collagen I and III levels, which were restored after ECH treatment. Contrary to the results of the histological analysis, the expression levels of *Col1a1, Col1a2,* and *Col3a1* increased in the OVX + ECH group; however, the downregulation in the expression of these procollagen molecules was not observed in the OVX group. 

To address these discrepancies, we evaluated MMP expression. MMPs belong to a family of enzymes involved in the turnover of the ECM. The expression of MMPs is stable in normal resting tissues but gets up-regulated during physiological and pathological stress and tissue repair. The expression of MMPs is reflective of the amount of collagenase and was found to be increased in the OVX group and suppressed in the OVX + ECH group. These findings may be related to the lower deposition of collagen I and III in the vocal fold lamina propria, as higher levels of collagenase in the OVX group resulted in the degradation of collagen. The results revealed that the most significant ECM component among the SHAM, OVX, and OVX + ECH groups was collagen. Thus, the ECM component that exerted maximum effects on the vocal fold in the OVX group was collagen and that ECH treatment modulated collagen expression and remodeled the lamina propria of the vocal fold by increasing collagen synthesis and decreasing collagen degradation. In contrast, hyaluronic acid concentration and elastin levels were not significantly different in all groups and ECH treatment had no influence on the expression level of hyaluronic acid and elastin until 6 weeks after ovariectomy. According to our previous study, hyaluronic acid concentration and elastin levels significantly decreased at 12 weeks after ovariectomy [6]. Thus, collagen synthesis and degradation seem to be the most important factors controlling early voice change caused by menopause.

**Figure 7:**
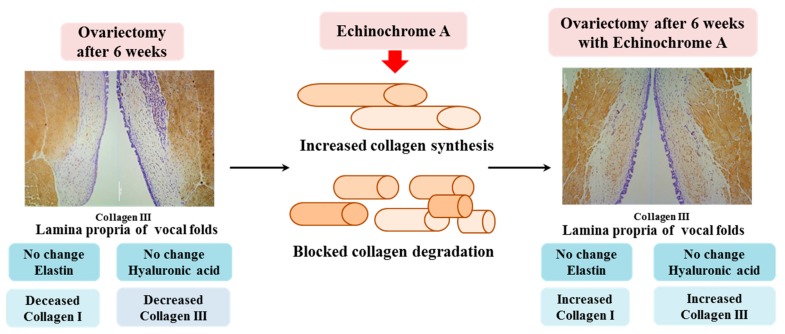
Summary of the current study. To summarize, hyaluronic acid and elastin were unchanged in the lamina propria of the vocal fold from the OVX group. Collagen I and III levels were significantly decreased in the lamina propria of the vocal fold in ovariectomized rats. However, ECH treatment increased the synthesis of collagen and decreased its degradation. We observed changes in several ECM-related genes in the OVX group after estradiol deficiency and ECH was shown to improve the altered expression of ECM components in the OVX group. Thus, the vocal fold is an estradiol-sensitive target organ and ECH may have protective effects on the ECM of vocal folds in ovariectomized rats.

Thus, the expression levels of hyaluronic acid and elastin were unchanged in the lamina propria of the vocal fold from the OVX group. Collagen I and III levels were significantly decreased in the lamina propria of the vocal fold in ovariectomized rats. However, ECH treatment increased the synthesis of collagen and decreased its degradation. We observed changes in several ECM-related genes in the OVX group after estradiol deficiency and ECH was shown to improve the altered expression of these genes in the OVX group (Figure 7). Thus, the vocal fold is an estradiol-sensitive target organ and ECH may have protective effects on ECM of vocal folds in ovariectomized rats.

## 4. Material and Methods

### 4.1. Animals

Forty-five female Sprague–Dawley rats, aged nine weeks, were used in this study (Samtako, Osan, Korea). Each group was weight-matched at the beginning of the study. After a week for acclimatization, rats were randomly divided into three groups. Group I (*n* = 15, sham-operated rats, called\SHAM), group II (*n* = 15, ovariectomized surgery rats, called OVX), and group III (*n* = 15, ovariectomized surgery rats treated with ECH, called OVX + ECH). Rats were sacrificed 6 weeks after ovariectomy. Six rats were used for histological study and nine rats were used for qPCR. All rats were maintained on a light/dark cycle of 12 h and provided rat chow with water ad libitum in a pathogen-free room. Animal care and research protocols were based on the principles and guidelines adopted by Guide for the Care and Use of Laboratory Animals (National Institutes of Health publication). This study was approved by the Review Board of Pusan National Hospital (IRB No.2018-04-017) and informed consent was waived. 

### 4.2. Echinochrome A

Echinochrome A is insoluble in water and, therefore, it is used for medical purposes in the form of injection, registered in Russia as the drug Histochrome (P N002363/02). Histochrome, trade name soluble echinochrome A sodium salt, does not contain other components. We used Histochrome, containing 0.2 mg/mL echinochrome A, produced by Pacific Institute of Bioorganic Chemistry, Far East Branch of the Russian Academy of Sciences.

### 4.3. Establishment of the Ovariectomized Rat Model

The rats were anesthetized using isoflurane inhalation (3% dissolved in oxygen). The ovariectomy (OVX) was as follows: rats were anesthetized and an incision made at the midline of the abdomen with the bilateral ovaries being revealed. In the OVX group, the ovaries were ligated and cut off bilaterally followed by the closure of the abdominal cavity. In the Sham operations group (SHAM), OVX surgery was performed by exposing the ovaries without the excision of ovaries. After ovariectomized surgery, rats were intraperitoneally injected with ECH at 10 mg/kg three times a week during 6 weeks (OVX + ECH).

### 4.4. Plasma Estradiol Analysis

Concentrations of estradiol in serum were measured by rat-specific estradiol enzyme-linked immunosorbent (ELISA) assay plates coated with a biotin-conjugated binding protein kit purchased from Calbiotech (Spring Valley, CA, USA). A cardiac puncture was performed and the blood spun at 3000 rpm for 30 min. Plasma was separated from the blood collected during exsanguination, immediately frozen in liquid nitrogen, and then stored at −80 °C.

### 4.5. Histology and Morphometric Analysis

The larynx was isolated from each rat and prepared for fixation overnight in 4% formalin. We used an automatic tissue processor for paraffin embedding (Leica, Wetzlar, Germany), TP1020, semi-enclosed benchtop tissue processor) and dispensing (Leica, Wetzlar, Germany) EG1150H, heated paraffin embedding module). Cross-sections (8 µm thick) were placed on glass slides and sections were prepared for immunohistochemistry, Verhoeff–Van Gieson elastin staining and Alcian blue staining. For staining analyses, slides were de-paraffinized with xylene and then hydrated through a series of washes in 100%, 85%, 75%, and 50% ethanol and finally water. We selected proper middle portions of lamina propria tissue for representative figures use undertaken at 200X pictures by light microscope (Leica, Wetzlar, Germany) DM4000/600M, versatile upright microscope for materials analysis). For quantitative analyses of collagen I, collagen III, elastin, and hyaluronic acid expression, we measured positively stained areas through image analysis using a system composed of a light microscope. Morphometric determination was undertaken at 40X pictures from the whole part of the lamina propria using the morphometric method, that used an image analysis system program composed of a light microscope (Leica, Wetzlar, Germany) Basic LAS V3.8 software). For the immunohistochemical analysis of vocal fold lamina propria, three non-overlapping areas were analyzed at 400X and a total of nine areas were analyzed in each section. Results were expressed as stained area per total area in micrometers squared. Data were expressed as medians and ranges.

### 4.6. Verhoeff–Van Gieson Elastin Staining

The Verhoeff–Van Gieson elastin staining method was performed to identify atrophy of elastic tissues. The sections were first placed in an iron hematoxylin solution for 10 min and then rinsed in distilled water and differentiated in 2% ferric chloride. After rinsing in distilled water and placing in 95% alcohol, the samples were counterstained with Van Gieson solution (Sigma Aldrich; St. Louis, MO, USA) for 5 min. The samples were then dehydrated in graded alcohol and then cleared in xylene and mounted.

### 4.7. Alcian Blue Staining

Alcian blue staining and a hyaluronidase digestion technique were performed to identify hyaluronic acid. Duplicate sections of the control samples were incubated in hyaluronidase (Sigma Aldrich, St. Louis, MO, USA) at 37 °C for one hour. After the wash, sections with and without hyaluronidase digestion were stained with Alcian blue stain (pH 2.5) for 30 min. Sections were washed and counterstained with nuclear fast red stain. Areas containing hyaluronic acid remained unstained in digested slides and stained blue in undigested slides.

### 4.8. Immunohistochemistry

De-paraffinized sections were washed with phosphate-buffered saline (PBS) and blocked for 1 h at room temperature with 2% bovine serum albumin (BSA) containing 0.3% Triton X-100 in PBS. They were then incubated for 24 h at 4 °C with the following primary antibodies: anti-estrogen receptor β (200 ug/mL) (Santa Cruz Biotechnology, Dallas, TA, USA), anti-collagen I, and anti-collagen III (1:400) (Abcam, Cambridge, UK). After the primary antibody was removed by rinsing, sections were incubated with secondary antibodies for 1 h at room temperature. The following goat-anti rabbit secondary antibodies (1:1000) (ENZO Biochem, NY, USA) were used for double-staining with DAB (3,3-diaminobenzidine) staining. Incubation with phosphate-buffered saline supplemented with 1% bovine serum albumin instead of the primary antibody served as a negative control.

### 4.9. Quantitative PCR

To confirm mRNA expression, we isolated whole lamina propria of the vocal fold with a syringe needle under microscope and used the real time PCR method. Tissue RNA was extracted using the TRIzol system (Life Technologies, Rockville, MD, USA). A reverse transcription kit (Applied Biosystems, Foster City, CA, USA) was used to perform reverse transcription according to the manufacturer’s protocol. Quantitative PCR was performed according to the SYBR ® Green PCR protocol (Applied Biosystems, Foster city, CA, USA). Each sample was tested in quintuplicate. Reaction conditions were: 10 min at 95 °C (one cycle); 10 s at 95 °C; and 30 s at 60 °C (40 cycles). Final primer concentration was 0.1 μM and sequences can be found in Table 1. Gene-specific PCR products were continuously measured by an ABI PRISM 7900 HT Sequence Detection System (PE Applied Biosystems, Waltham, CT, USA). All the primers used for qPCR analysis had been designated using Primer Express software 1.5 (Applied Biosystems, Foster city, CA, USA) and synthesized by Invitrogen. (Invitrogen Life Technologies, Carlsbad, CA, USA). Normalization consisted of using the differences between the cycle thresholds (delta CT) and the expression level for *Rn18s* to calculate the delta CT/target gene delta CT ratio. Delta/delta CT corresponds to the differences between the delta CT the internal control gene.

### 4.10. Statistical Analysis

Unless otherwise noted, all quantitative data were reported as the mean standard error of the mean from at least three parallel repeats. One-way analysis of variance was used to determine significant differences between groups in which *p* < 0.05 was considered statistically significant.

## 5. Conclusions

We observed changes in genes expression associated with ECM production and degradation in the lamina propria of the vocal fold after the induction of menopause by ovariectomy in a rat experimental model. The changes in the genes related to the generation and degradation of ECM were observed after the administration of ECH. This study suggests that the antioxidant properties of ECH may have protective effects on the vocal fold caused by lamina propria ECM deposition. These results indicate that the vocal fold is an estradiol-sensitive target organ and that ECH may have protective effects on the ECM of vocal folds in ovariectomized rats.

## Figures and Tables

**Table 1:** Sequence of primers.

*Esr2a* (estrogen receptor βI)	Forward	GCTTCGTGGAGCTCAGCCTG
	Reverse	AGGATCATGGCCTTGACACAGA
*Esr2b* (estrogen receptor βII)	Forward	GAAGCTGAACCACCCAATGT
	Reverse	CAGTCCCACCATTAGCACCT
*Has1* (hyaluronic acid synthase 1)	Forward	CCACTGCACATTTGGGGATG
	Reverse	GAATAGCATCTGGAGCGCGA
*Has2* (hyaluronic acid synthase 2)	Forward	ACTGGGCAGAAGCGTGGATTATGT
	Reverse	AACACCTCCAACCATCGGGTCTTCTT
*Has3* (hyaluronic acid synthase 3)	Forward	GCACCATTGAGATGCTTCGG
	Reverse	TACCTCACGCTGCTCAGGAA
*Eln* (tropoelastin)	Forward	TTCTGGGAGCGTTTGGAG
	Reverse	CCTTGAAGCATAGGAGAGACCT
*Cela1* (chymotrypsin-like elastase family member 1)	Forward	TCCTAGGAGCCAGGCCATT
	Reverse	GGGTAGATAGGAGAAAGTCCAAACC
*Col1a1* (collagen, type I, alpha 1)	Forward	AGTCCATCTTTGCCAGGAGAACCA
	Reverse	CGGCAGGACCAGGAAGACC
*Col1a2* (collagen, type I, alpha 2)	Forward	CCGAGGCAGAGATGGTGTT
	Reverse	GCAGCAAAGTTCCCAGTAAGA
*Col3a1* (collagen, type III, alpha 1)	Forward	ACTGACCAAGGTAGTTGCATCCCA
	Reverse	CCAGGGTCACCATTTCTCC
*Mmp1* (matrix metallopeptidase 1)	Forward	ATGAGACGTGGACCGACAAC
	Reverse	TGAGTGAGTCCAAGGGAGTG
*Mmp2* (matrix metallopeptidase 2)	Forward	GTC ACT CCG CTG CGC TTT TCT CG
	Reverse	GAC ACA TGG GGC ACC TTC TGA
*Mmp8* (matrix metallopeptidase 8)	Forward	CAGACAACCCTGTCCAACCT
	Reverse	GGATGCCGTCTCCAGAAGTA
*Mmp9* (matrix metallopeptidase 9)	Forward	CGGAGCACGGGGACGGGTATC
	Reverse	AAGACGAAGGGGAAGACGCACATC
*Rn18s* (18s RNA)	Forward	AACCCGTTGAACCCCATT
	Reverse	GGGCAGGGACTTAATCAACG
